# Integrative brain omics approach reveals key role for sn-1 lysophosphatidylethanolamine in Alzheimer's dementia

**DOI:** 10.21203/rs.3.rs-3973736/v1

**Published:** 2024-02-27

**Authors:** Eric Ortlund, Chih-Yu Chen, Kristal Maner-Smith, Manoj Khadka, Jun Ahn, Xueyun Gulbin, Anna Ivanova, Eric Dammer, Nicholas Seyfried, David Bennett, Ihab Hajjar

**Affiliations:** Emory University School of Medicine; Emory integrated metabolomics and lipidomics core; Emory University School of Medicine; Emory integrated metabolomics and lipidomics core; Emory integrated metabolomics and lipidomics core; Emory University,; Emory integrated metabolomics and lipidomics core; Emory University; Emory University School of Medicine; Rush University Medical Center; University of Texas Southwestern

## Abstract

The biology of individual lipid species and their relevance in Alzheimer’s disease (AD) remains incompletely understood. We utilized non-targeted mass spectrometry to examine brain lipids variations across 316 post-mortem brains from participants in the Religious Orders Study (ROS) or Rush Memory and Aging Project (MAP) cohorts classified as either control, asymptomatic AD (AAD), or symptomatic AD (SAD) and integrated the lipidomics data with untargeted proteomic characterization on the same individuals. Lipid enrichment analysis and analysis of variance identified significantly lower abundance of lysophosphatidylethanolamine (LPE) and lysophosphatidylcholine (LPC) species in SAD than controls or AAD. Lipid-protein co-expression network analyses revealed that lipid modules consisting of LPE and LPC exhibited a significant association to protein modules associated with MAPK/metabolism, post-synaptic density, and Cell-ECM interaction pathways and were associated with better antemortem cognition and with neuropathological changes seen in AD. Particularly, LPE 22:6 [sn-1] levels are significantly decreased across AD cases (SAD) and show the most influence on protein changes compared to other lysophospholipid species. LPE 22:6 may be a lipid signature for AD and could be leveraged as potential therapeutic or dietary targets for AD.

## Introduction

Alzheimer’s disease (AD) is the most prevalent cause of dementia worldwide^1^ and is characterized by the combined presence of β-amyloid plaques and tau neurofibrillary tangle deposition^2^. AD pathogenesis is complex and may involve the disruption of many molecular pathways related to protein-protein interaction as well as protein-lipid interactions^2–6^. The prevalence of cognitive impairment increases exponentially with advanced aging making this the most important risk factor for AD^2^. Given the complexity of aging and multifactorial pathology in AD, phenotypic heterogeneity in AD ranges from asymptomatic AD (AAD), to mild cognitive impairment (MCI), to dementia (specifically dementia due to AD, which we subsequently refer to as symptomatic AD, SAD)^7^; however, the ability to categorize and diagnose AD via observation of biochemical changes in the brain remains elusive. To gain insight into the molecular networks in AD brains, we aimed at conducting non-targeted lipidomics on the post-mortem brains from the Religious Orders Study (ROS) or Rush Memory and Aging Project (MAP) and integrated our lipidomics data with the matching non-targeted proteomic data obtained by our team^8^ on the individuals from the same cohort, with further integration of rich antemortem clinical and post-mortem pathological traits for the ROSMAP individuals.

The brain is the second-most lipid-rich organ after adipose tissue^9^ and lipid trafficking is essential for maintaining physiological functions such as membrane formation, neurotransmitter transport, and energy production^10–12^. Lipids constitute 50–60% of the brain dry weight^13^ with neural membranes and myelin comprised largely of cholesterol, sphingolipids, and glycerophospholipids^14^. Increased cholesterol levels in the brain directly regulate γ-secretase activity^15^ and sphingolipids, including ceramides, sphingomyelin, and glycosphingolipids (GSL or HexCer), are directly involved in amyloid precursor protein (APP) metabolism, leading to β-amyloid accumulation and aggregation^16^. β-amyloid oligomers induce the hydrolysis of glycerophospholipids such as phosphoinositide (PI) and phosphatidylcholine (PC) by phospholipase C (PLC) and PLD2/PLA2, respectively, and further elevate calcium release from the endoplasmic reticulum (ER) as well as inflammatory prostaglandin production, resulting in synaptotoxicity^15,17^. Recently, targeted and non-targeted lipidomic investigations of AD have demonstrated unbalanced lipid homeostasis associated with brain pathology in biological fluids and brain tissues^7,18^. Although abnormal lipid metabolism has been known as a critical contributor to AD pathogenesis, lipid profiles of the AD brain and how they relate to AD pathophysiology remain incompletely explored.

Recent advancements in liquid-chromatography coupled with high-resolution mass spectrometry permit deep interrogation, identification, and quantification of the lipidome. We utilized lipidomic methodology to compare the lipid profiles of post-mortem dorsolateral prefrontal cortex (DLPFC) samples from cases of control, asymptomatic AD (AAD), and symptomatic AD (SAD) within the ROSMAP. We identified differentially abundant LPE species (LPE 20:4 and LPE 22:6) between groups that are highly associated with AD case classifications. Using a multivariate, module-based approach, the lipid modules most strongly associated to AD clinical traits were enriched in LPE only or a mixture of LPE and LPC, and were highly correlated to previously reported AD-relevant protein modules including the following: MAPK/metabolism, Post-synaptic density, and Cell-ECM interaction^8^. Our results highlight the importance of lipid-protein interaction networks in the AD pathogenesis and provide a robust framework for future multi-omics studies on AD brains.

## Results

### Overview of Workflow

**Figure 1:**
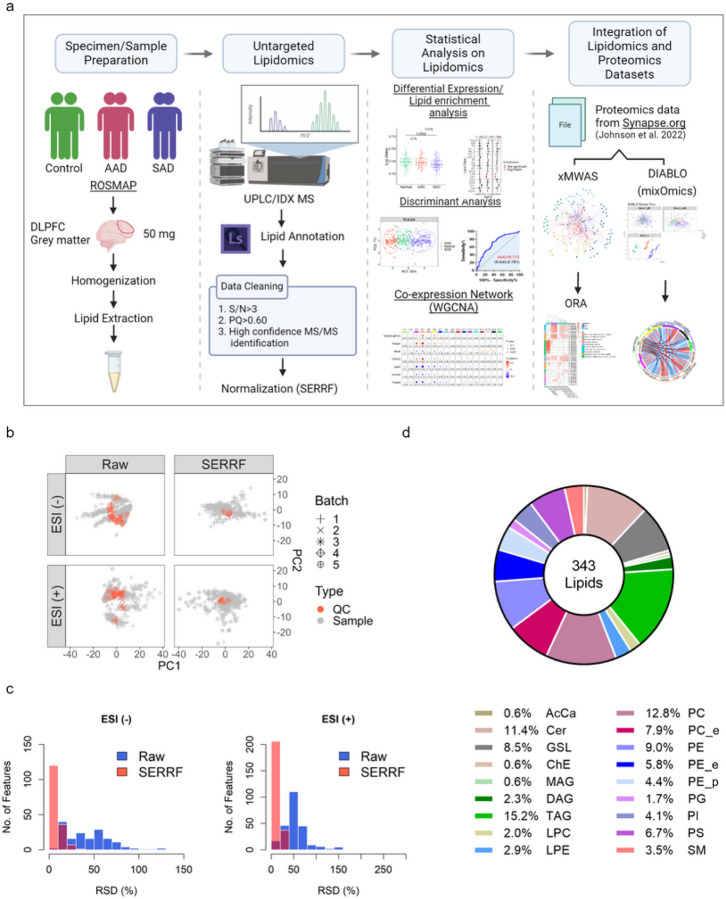
Overview of DLPFC dataset of ROSMAP. (a) Graphic illustration of the pipeline to acquire DLPFC lipidome data as input for integrative multi-omics analysis. (b) Principal component analysis (PCA) plots were obtained before (left) and after (right) SERRF normalization for human brain lipidomics data acquired in negative (-) and positive (+) electrospray mode (ESI). Pooled QC samples and human brain samples are represented as red and black colors, respectively. (c) The distribution of sample RSD (%) presented as histrograms. Raw- and SERRF corrected-data are highlighted as blue and red, respectively. (d) Pie chart showing the relative lipid class composition of all ROSMAP brain samples. Abbreviation: AcCa: Acyl carnitine; Cer: Ceramide; ChE: Cholesterol ester; GSL: Glycoshingolipids; LPC: Lysophosphatidylcholine; LPE: Lysophosphatidylethanolamine; MAG: Monoacylglyceride; PC: Phosphatidylcholine; PC_e: Plasmanyl PC; PE: Phosphatidylethanolamine; PE_e: Plasmanyl PE; PE_p: Plasmenyl PE; PG: Phosphatidylglycerol; PI: Phosphatidylinositol; PS: Phosphatidylserine; DAG: Diacylglyceride; SM: Sphingomyelins; TAG: Triacylglyceride.

Our overall strategy was to discriminate significant differences in DLPFC-derived lipids between groups of AAD or SAD individuals relative to controls, and to identify the biological pathways associated with these significant lipids using integrated omics methods. Briefly, we processed 316 autopsied tissues from gray matter of DLPFC in 92 controls, 77 AAD subjects, and 147 SAD subjects. A non-targeted UPLC-MS/MS assay was applied to obtain a broad coverage of the brain lipidome through the combination of high-resolution accurate-mass and ddMS^2^ data acquisition. The raw data were processed and all mass spectra were manually investigated through LipidSearch, yielding a total of 2200 annotated lipids (including deuterium-labeled lipids and internal standards). 343 among these annotated lipids met the data filtering criteria and were used for batch correction by SERRF R package (Fig. S1, and validated lipids are listed in Table S3.). To determine significant abundance differences in lipids among control and AD groups, the normalized lipid profiles were used for single-omic statistical analysis. Lipid modules were generated by WGCNA and further integrated with proteomic modules obtained from Johnson *et al*. (2022)^8^ using DIABLO. Figure 1a illustrates this study design, with detailed information given in methods.

### Demographics of Participants

The basic characteristics of brain samples are summarized in Table 1. Study subjects from these three groups had similar education levels and the majority of samples in each group were female. Relative to controls, both AD groups had higher global pathology, β-amyloid, tangles, and Braak, and lower scores for CERAD as well as Reagan (P < 0.05), where the last 2 metrics are inverted scores as recorded for the ROSMAP compared to standard versions of these scores. AAD and SAD groups were older on average (P < 0.001) than controls, and importantly SAD had significantly lower global (all-domain) cognitive performance (GlobalCongFunc) (P < 1.0E-7), as expected.

### Differential Profiles of Lipid Classes in Brain among the Three Groups

**Figure 2:**
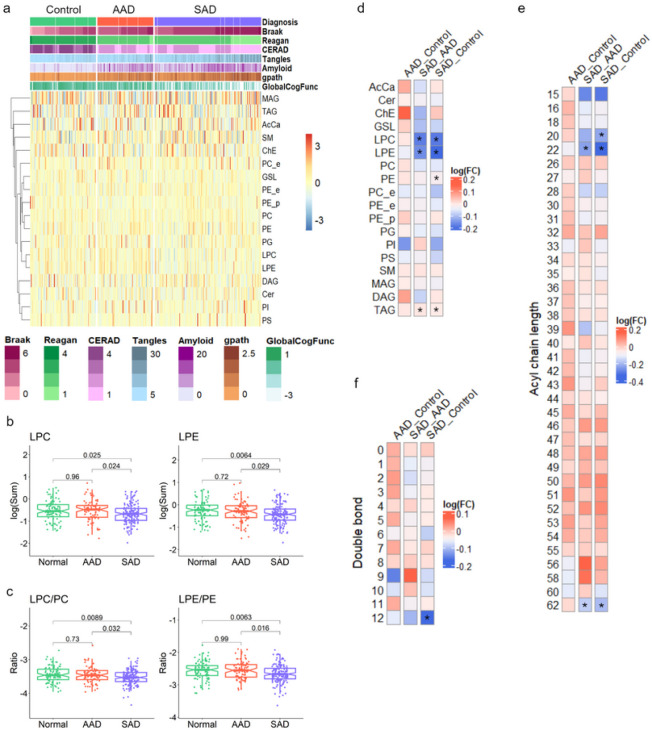
Characteristics of AD clinical traits and the non-targeted lipidome. (a) The abundance of each lipid class was converted to a Z score and is represented in the heat map (blue for low expression and red for high expression). The heat map shows lipid classes differentially expressed across AD clinical traits and the three case classifications. (b-c) Boxplots of median values, all sample points for each individual, and quartiles below and above the median within the three case classes for LPE, LPC, the ratios of LPE/PE and LPC/PC. One-way ANOVA with Tukey’s multiple comparison test across the three case classifications was performed. (d-f) Heat map matrix of pairwise comparison for lipid class, total acyl chain length, and unsaturation (double-bond) levels shown as the distribution of log_2_ fold change between pairs of case classes. Asterisk (*) indicates a significant enrichment (p<0.05) using the LSEA method.

QC samples’ raw data showed systematic drifts in both positive and negative electrospray modes, and after SERRF normalization, QC distribution was largely tightened to one cluster (Fig. 1b). SERRF-normalized data showed consistently reduced RSD and a large increase in the number of lipids with less than 50% of RSD compared to raw data (Fig. 1c). In total, 343 normalized, annotated, lipids were not strongly influenced by age, gender, education, post-mortem interval (pmi), and race, and were used for downstream analysis (MANOVA test, P > 0.05). A total of 15 lipid classes were measured, and the first and second in net abundance in brain were triacylglycerides (TAG) (15.2%) and phosphatidylcholine (PC) species (12.8%), respectively (Fig. 1d). As depicted in Fig. 2a, a heatmap displaying an overview of lipid classes reveals larger fluctuation across sample groups in z scores of monoglycerides (MAG), TAG, acyl carnitines (AcCa), and sphingomyelins (SM) compared to phospholipids. Of 15 lipid classes, the SAD diagnosis group had significantly lower levels of lyso-phospholipids including lysophosphatidylethanolamine (LPE) and lysophosphatidylcholine (LPC) species compared to the AAD and control groups (p < 0.05). Phosphatidylethanolamine (PE) abundance trended lower in the AAD group (P = 0.68) and was significantly lower in the SAD group (P = 0.019) relative to the controls. PC was detected at similar levels among the three groups, resulting in a slightly lower ratio of PC to PE in controls (Fig. S2). Interestingly, the ratios of LPE to PE and LPC to PC were significantly reduced in the SAD group compared to either control or AAD groups, indicating a decrease of potential for hydrolysis of phospholipids to lyso-phospholipids in SAD (Fig. 2c). Overall, the phospholipid classes were at similar levels in control and AAD groups, but trended lower in SAD (Fig. S2). By dissecting lipid classes into subclasses by desaturation status and acyl chain length, the lowest mean abundances of phospholipids including PC, PE, PG, PS, PI, LPC, and LPE at either the level of desaturation or that of acyl chain length were mostly observed in the SAD group (Fig. S3, S4a and S5a). The TAG desaturation levels with double bonds ranging from 1 to 7 and acyl chain net length ranging from 50 to 58 carbons tended to be higher in SAD than controls (Fig. S3, S4b, S5b). In comparison to controls, phospholipids with acyl chain lengths of 20 carbons (Fig. 4Sa) and TAG with 44, 56, and 58 net carbons were significantly lower in AAD, while TAG with 53 carbons was higher in AAD (Fig. 4Sb). Concerning desaturation status, AAD showed levels similar to SAD for TAG containing 0–4 double bonds while AAD had the lowest values compared to the other groups considering TAG containing 5–10 double bonds, except TAG containing 9 double bonds (Fig. S5b). Lipid set enrichment analysis (LSEA), revealed LPE, LPC, and TAG classes were significantly different between SAD and AAD, and comparing SAD versus control groups. The PE class showed significant enrichment only in the comparison of SAD versus control groups (Fig. 2d). Leading-edge class members that drive significant enrichment of differentially abundant lipid classes are listed in supplementary Table S4. Performing lipid set enrichment analysis (LSEA) for differentially quantified lipids of specific net acyl chain length, lipid subsets with 22 and 56 carbons were significantly enriched in SAD comparing to either AAD or controls. Lipids with 20 carbons only showed significant enrichment comparing SAD to the control group (Fig. 2e). Lipids with a total acyl chain length of 22 carbons (LPE 22:6, LPC 22:6, LPE 22:5, and LPE 22:4) and of 20 carbons (LPC 20:4, LPE 20:4, and LPE 20:3) showed significant enrichment in SAD, and were mainly from LPE and LPC classes. TAG species containing oleic acid (C18:1) such as TAG 18:0_18:1_20:4, TAG 18:1_18:1_20:4, TAG 20:1_18:1_18:2, and TAG 16:0_18:1_22:6, are driving this net-56-carbon-lipid enrichment (Table S5.). The lipid subset with 12 double bonds was found to be significantly enriched in SAD versus controls (Fig. 2f). Intriguingly, DHA (22:6), containing 6 double bonds, is mainly attributed to the leading-edge of this subset, which also includes PE 22:6_22:6, TG 18:0_22:6_22:6, PG 22:6_22:6, PS 22:6_22:6, and TG 16:0_22:6_22:6 (Table S6). At lipid class level, the most prevalent changes including in abundance and enrichment significance among lipids with acyl chain length of 20 and 22 carbons changing between SAD and control groups were found to be LPE or LPC, suggesting an important role of brain lyso-phospholipids in AD phenotypes (Table S5.).

### LPE 22:6 [sn-1] is Differentially Abundant across the Cohort

**Figure 3:**
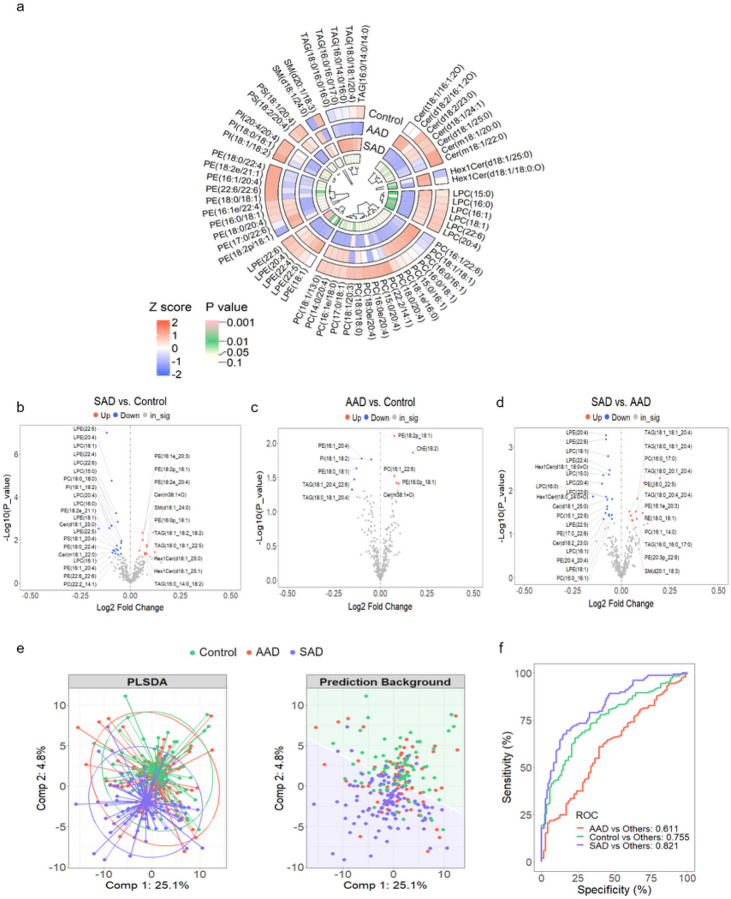
Identification of significant differentially abundant lipid species across the three groups. (a) The abundance of 59 annotated lipid species were converted to Z scores and represented in a circos plot (blue for low expression and red for high expression above the mean, white). The innermost track shows color based on non-parametric Kruskal–Wallis tests across the three groups and the color gradient indicates significance (P) from white for a raw P value=0.115 to pink for a raw P value<0.001. (b-d) Volcano plots showing log_2_ fold change versus −log(P_value) for pairwise comparisons, as calculated by the R package, limma. Lipid species demonstrating statistically significant differences in means between two case classes are highlighted in red for increased fold changes and in blue for decreased fold changes, with a significance level of P<0.05. (e) Supervised PLS-DA (sPLS-DA) analysis of lipidomic data with the first two principle components (Comp 1 and Comp 2) presented. Confidence ellipses surrounding core Controls, AAD, and SAD group points (left) and the prediction background (right) as analyzed by the R package, mixOmics. (f) ROC analysis and AUC from sPLS-DA with 2 PCs.

To understand the overall differences in lipid abundance among the three diagnosis groups, and to validate the results from Fig. 1, a circular heatmap was drawn to visualize averaged abundance of each lipid group after normalization. Based on a non-parametric Kruskal–Wallis test, 59 out of the 343 annotated lipids met the criteria of FDR < 0.7 (raw P value < 0.12) and their averaged abundance per group is shown (Fig. 3a, abundance of all lipid species is shown in Fig. S6.). LPE 22:6 is the only significantly differentially abundant lipid across the three groups (FDR = 0.02, raw P value = 5.07E-5). LPE 20:4 reached statistical significance for the raw P value, 5.76E-4 (FDR = 0.099). LPE 22:6 regiospecificity (sn-1 vs sn-2) of the glycerol backbone has not been specified in previous studies and the physiological function of LPE might be different based on this structural distinction. In our study, lysophospholipid regiospecificity was determined by manual inspection of MS/MS fragment data. The fragmentation pattern of phospholipids is well established, resulting in peaks from the loss of headgroup fragments from the intact species, as well as peaks from each fatty acyl anion^19,20^. Lyso-phospholipids have one fatty acid and one hydroxyl group covalently bonded to the phosphoglycerol backbone. Polyunsaturated fatty acids are predominantly located at the sn-2 position of phospholipids; liberation of PUFA from membrane phospholipids by PLA_2_ results in lyso-phospholipids with a fatty acyl chain at the sn-1 position^21^. Fragmentation in the mass spectrometer at the sn-2 position of lyso-phospholipids with a polyunsaturated fatty acid, such as docosahexaenoic fatty acid (DHA; 22:6) is shown in Fig. S7d-e. The mass spectrum shown are peaks resulting from the intact lipid as well as the fatty acyl anion, m/z 524 and m/z 327, respectively. Upon fragmentation, DHA rapidly loses carbon dioxide from the carboxy-terminus, resulting in a peak at m/z 283. When DHA is at the sn-1 position of lyso-phospholipids (Fig. S7f-g), a peak at m/z 196 is also present^20^. This peak is reflective of the remaining hydroxy group on the newly fragmented lysophospholipid. A peak at m/z 196 is also present in commercially available internal standard sn-1 18:1D7 LPE (Fig. S7b-c). For additional confirmation of positional isomers of LPE species, it has been previously reported that LPE [sn-2] isomer elutes prior to LPE [sn-1] on C18 chromatography^22^. This trend is appreciated in Fig. S7d-g; here the predominant species is LPE 22:6 [sn-1].

Using differential expression analysis, we identified magnitude of significant changes in lipid species between the two groups within these three clinical categories. The volcano plots highlight species with raw P value below 0.05 between groups, as presented in Fig. 3b–d. Relative to controls, a total of 32 lipids (for SAD) and 10 lipids (for AAD) were found to have apparent fold change. Intriguingly, lyso-phospholipids including LPE 22:6, LPE 22:5, LPE 20:4, LPC 22:6, LPC 20:4, LPC 18:1, and LPC 15:0, exhibited significant differences between SAD and control groups, and also between SAD versus AAD, but remained at a similar level comparing AAD earlier in the disease course to the control group. Between AAD and control groups, notable changes of annotated lipids were mostly found within phospholipids. To further investigate whether AD classification can be performed based on lipid profile, supervised partial least squares discriminant analysis (PLS-DA) was performed using the sparse PLS-DA algorithm from the mixOmics R package. The first two principal components, PC1 and PC2 explained 25% and 5% of the total variance in lipid profile, respectively, and were used for plotting PLS-DA, and were also used to dichotomize the individual case samples for ROC analysis. The PLS-DA result exhibited a poor classification across three groups (Fig. 3e, left panel), but interestingly, the clustering centers and background prediction areas between control and SAD groups were clearly separated (Fig. 3e, right panel). The ranking PLS-DA analysis revealed that LPE 22:6 has the highest eigenvalue of PC1 and mainly contributes to the discrimination of controls from other groups (Fig. S8a). The sPLS-DA algorithm, including PC1 and PC2 exhibited enhanced classification accuracy (Fig. 3f), with an increased AUC under the ROC curve to 0.821. This improvement was observed specifically in the comparison of SAD vs. all other non-SAD cases (Wilcoxon test P = 0.0E0). However, the accuracy was incrementally lower at 0.755 when distinguishing control vs. all other cases (Wilcoxon test P = 1.4E-11), and further reduced to an AUC of 0.611 for the classification of AAD compared to other cases (Wilcoxon test P = 5.5E-03). The sPLS-DA algorithm model yielded a substantially higher AUC than any individual annotated lipid, such as LPE 22:6 which had the highest individual species AUC value (AUC for SAD versus Control cases of 0.631, P = 0.0016) (Fig. S8b). Our brain lipidomic results indicate that the most robust overall changes were found between control cases and those of the SAD group, and the most significant species changing in this cohort was LPE 22:6 [sn-1].

### Construction of a Lipid Co-expression Network

**Figure 4:**
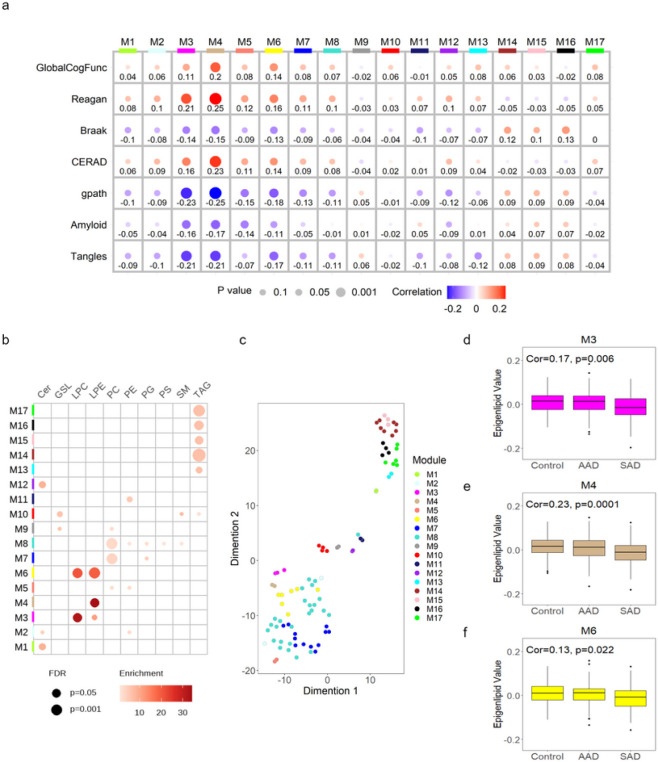
Network analysis of the ROSMAP DLPFC lipidome. Lipid levels in DLPFC from Controls, AAD, and SAD participants measured by UPLC-MS and analyzed by WGCNA and for differential abundance. (a) A lipid correlation network consisting of 17 lipid modules generated from 343 lipids in the ROSMAP cohort (n=314 DLPFC case samples, 2 outliers were removed). Module eigenlipids, which represent PC1 of the lipid abundance within each module, are correlated with AD clinical traits including GlobalCogFunc, β-amyloid, Braak, CERAD (inverted scale), Tangles, Reagan (inverted scale), and global pathology (gpath). A bubble heatmap shows the correlation and P value between lipid modules and clinical traits, in which the size of bubbles indicates P value (the larger the bubble size, the lower the p values are) and the color of bubbles indicates either positive (red) or negative correlation (blue). The numbers underneath bubbles are correlation coefficients. ME correlations and P values were performed using Pearson correlation and Student asymptotic P value for given correlations in the WGCNA R package. (b) The bubble heat map showing the lipid class enrichment in each of 17 lipid modules. The size of the bubble indicates the FDR (the larger the bubble size, the lower the P value.), and the color the bubble indicates the overlap (enrichment) of lipid class in each module. (c) Dimensionality reduction and visualization by t-SNE analysis was applied to all the lipids within each lipid network module. (d-f) Module eigenlipid levels by AD case status for 3 lipid modules that had significant correlations to all AD clinical traits in (a) were assessed by Kruskal-Wallis test.

The log_2_-transformed data of 343 lipids were used to generate a lipid co-expression network using the WGCNA algorithm. As depicted in Fig. 4a, the resulting network consisted of 17 lipid modules ranging in size from 2 lipids to 26 lipids across 314 cases analyzed, after excluding outliers. Detailed information including module membership (module epigenlipid values, MEs) and P values are shown in supplementary Table S7. t-distributed stochastic neighbor embedding (t-SNE) analysis was applied to lipid species within each AD lipid network by their kME values and no overlapping was observed, indicating that the lipid modules identified by WGCNA are robust and independent (Fig. 4c). To assess whether a given co-expression network module was related to AD clinical traits, we correlated the MEs to GlobalCogFunc, global pathology (gpath), β-amyloid, tangles, Reagan, CERAD, and Braak. We also correlated these MEs to binary AD case classification (control, SAD, and AAD), determined by a combination of neuropathological and cognitive metrics as described in the method section. Three lipid modules (M3, M4, and M6) were found to be significantly associated with all AD clinical traits (P < 0.05). These three modules have strong correlation with their intramodular LPE, and in some cases, LPC, class members as indicated by high intramodular kME for these members (Fig. 4b). Specifically, the magenta M3 module is enriched in LPC 22:6, LPC 15:0, and LPE 22:5; the tan M4 module is enriched in LPE 22:6 and LPE 20:4; the yellow M6 module is enriched in LPC 18:1, LPC 16:0 LPC 16:1, LPE 22:4, LPE 18:1, LPE 18:0, and LPE 18:1e. Module membership (MM) of each lipid species within the module was highly relevant and greater than 0.8. There was negative correlation to the AD binary case classification for relevant MEs, M3 (cor=-0.17; P = 0.006), M4 (cor=-0.23; P = 0.0001), and M6 (cor=-0.13; P = 0.022) (Fig. 4df). M14, M15, and M16, all enriched in different TAG species, were significantly associated with Braak (cor≥0.1) (Fig. 4a). We hypothesized that integration of these lipid modules of interest with proteomic modules across common cases of the same ROSMAP cohort generated by WGCNA could provide insight into biological processes, molecular functions, and cellular components altered in conjunction with the lipid profiles of these modules, either upstream, downstream, or coincidentally.

### Lipidome-Proteome Integration for Discrimination of Lipid-associated DLPFC changes in AD

**Figure 5:**
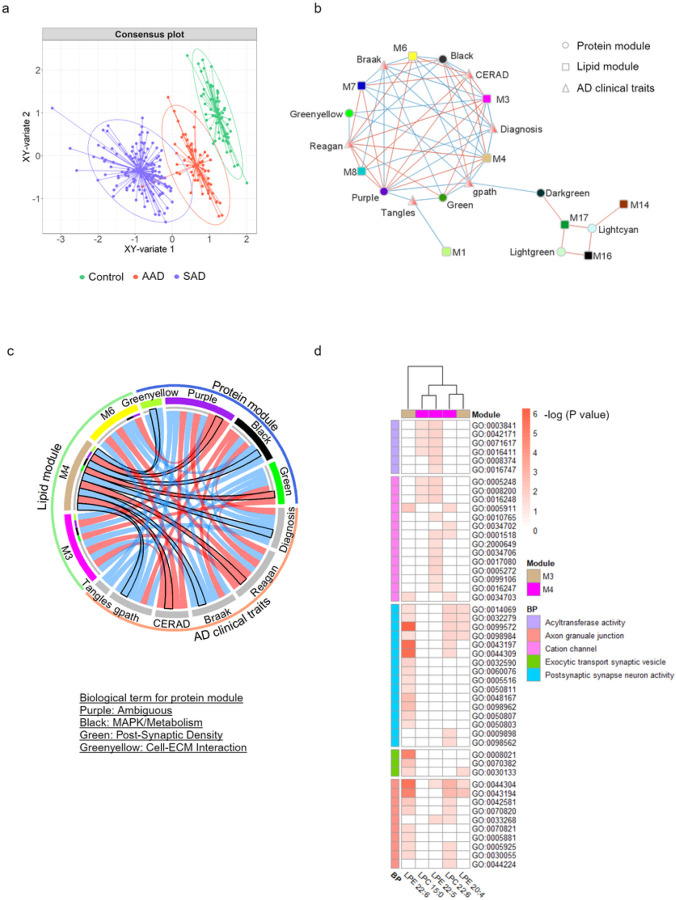
Lipid modules associated with protein modules relevant to AD neuropathology. DIABLO-processed data for lipid modules, protein modules, and clinical traits as input was used for multi-omics integration analysis. (a) A DIABLO consensus component plot was generated from the identified multi-omics biomarker panel: test samples (dots) were overlaid with 95% confidence ellipses calculated from the training data (ellipses). X variate indicated the average of the components from each dataset and Y-variate indicates different sample types, the three case classifications. (b) Relevance network showing the associations of the lipid network, protein network, and clinical traits at cutoff=0.7. This plot was generated using Cytoscape. (c) A chordDiagram represents the associations of three lipid modules containing LPE and LPC to the protein network and AD clinical traits. The lipid M3 module containing LPE 20:4 and LPE 22:6 is highlighted in a black outline. (d) The hub lipids from lipid modules M3 (LPC 15:0, LPC 22:6, and LPE 22:5) and M4 were further used for correlation analysis to protein expression by xMWAS. The proteins relevant to each hub lipid with correlation>0.6 at p<0.05 were applied to gene set enrichment analysis by the R package ClusterProfiler. The heat map of associations of each lipid species to Gene ontology (GO) terms and biological pathways. Enrichment for a given ontology was adjusted by Bonferroni correction and is shown by −log_10_(P value). P values of 0.05 and 10^−6^ refer to −log(P values) of 1.3 and 6, respectively.

A proteomic co-expression network was published by Johnson *et al*. in 2022 on the ROSMAP cohort DLPFC tissue samples^8^. Briefly, a total of 516 DLPFC tissue samples from ROSMAP^23^ and the Banner Sun Health Research Institute^24^, were analyzed by tandem mass tag mass spectrometry (TMT-MS), and a total of 8619 proteins were used to build a protein co-expression network using WGCNA. This network consisted of 44 proteomic modules and the biology represented by each module was determined using gene ontology enrichment for its constituent proteins. To identify a subset of lipid and protein modules that discriminate between the three case diagnoses in our study, we employed DIABLO^25,26^, a multi-block sPLS-DA approach. This method integrates multiple omics datasets using the input of modules (hereafter used interchangeably with MEs) from both the lipid and protein networks, along with the clinical traits common to both sets, thereby allowing for pairing of the networks. Using DIABLO’s model, although the averaged X-variates of the combined proteomic, lipidomic, and clinical datasets did not show clear discrimination among the three groups, control samples were evident as a separate cluster and could already be discriminated from most SAD samples (Fig. S9d). After including a Y-variate for the three case classes, clearer cluster separation was observed in a consensus plot of composite XY-variate axes (Fig. 5a). The clinical and proteomic data showed the highest discriminatory capacity and correlation (cor = 0.53). DIABLO analysis identified 5 lipid modules, 25 protein modules, and 5 clinical AD traits that distinguished SAD from the control group (Fig. S9e-g). To visualize the multi-omics integration results, a network plot was used to show the inter-connection of nodes with edges kept representing absolute correlation of more than 0.7 (Fig. 5b). Lipid modules M14, M16, and M17, consisting of TAG species, were highly correlated to protein modules for RNA splicing (lightgreen) and RNA binding (lightcyan); this 3-lipid module cluster was positively correlated to the post-synaptic density (darkgreen) protein module and negatively correlated with the clinical trait for global pathology. The M1 lipid module, consisting of ceramide species, was negatively correlated to the clinical trait for quantification of tangles, but not directly correlating to any protein module. Depicted in Fig. 5c, the M3 and M4 lipid modules were highly correlated to protein modules for MAPK/metabolism (black), a distinct post-synaptic density module (green), and cell-ECM interactions (greenyellow). We employed xMWAS network analysis to assess the individual correlation between lipid species extracted from lipid modules M3 and M4, and individual proteins. Subsequently, gene ontology (GO) enrichment analysis was utilized to elucidate the biology of the group of proteins strongly associated with individual lipid species. Consistently, LPE 22:6 [sn-1], which had a strong membership within the M4 lipid module, was highly associated with postsynaptic synapse neuron activity (Fig. 5d; the description of GO terms written in Table S8), and had the highest association with proteins that also associated with other lipid species (Fig. S10). Utilizing a proteomic dataset from iPSC-derived neurons^67^ from matched ROSMAP individuals (Control = 10, SAD = 5, AAD = 12), we examined the correlations between LPE 22:6 [sn-1] and protein expression. We found that LPE 22:6 [sn-1] is highly associated with Echinoderm microtubule-associated protein-like 4 (EML4), which is essential for the stabilization of microtubules (cor=-0.61, p = 0.00078; Fig. S13a). Based on our results, we demonstrated that LPE, specifically LPE 22:6 [sn-1], is strongly associated with post-synaptic neuronal function, and could be a potential biomarker for distinguishing SAD cases from control ones.

## Discussion

We measured non-targeted lipidomics on 316 post-mortem brain samples and employed a multi-omics network approach to integrate proteomic data available for the same brain region of the same brains. Our key findings are: lower abundance of total LPE and LPC in symptomatic stages of AD compared to control or asymptomatic cases. Although brain lipid composition differs between brain regions and shows dynamic changes during development and aging, overall, the deficiency of brain PE and PE plasmalogens are associated with AD^27–32^, and a decreased ratio of PC/PE inhibits γ-secretase activity, resulting in less β-amyloid formation^33^. Consistent with this observation, the abundance of plasmalogens, PC_e (p = 0.087) and PE_e (p = 0.26), trended lower in SAD vs controls (Fig. S2). The PC/PE ratio was slightly decreased in controls relative to the SAD group, in line with decreased BACE1 and APP (Fig. S2 and S12). Lipid class variation may be due to membrane remodeling, consistent with phospholipid composition redistribution rather than a dramatic change in lipid metabolism, which is rarely seen in the brain^34^. Also, previous studies demonstrated that oleic acid-enriched TAG accumulates in ependymal cells in both postmortem AD brains and a transgenic AD mouse model is associated with the suppressed regeneration and homeostasis of neural stem cells^35^. In our LSEA results, we found that oleic acid enriched TAG was significantly elevated in SAD versus control case DLPFC, possibly suggesting the involvement of oleic acid enriched TAG in AD. Targeted lipidomics showed a decreased ratio of PC/PE was also observed in controls compared to SAD and AAD (Fig. S10d). In general, controls tended to have slightly higher levels than other groups of phospholipids incorporating omega-3 and omega-6 polyunsaturated fatty acids (PUFA), including DHA (omega-3), DPA (omega-3), AA (omega-6), and LA (omega-6) (Fig. S10b-c). A decrease of total PUFA in phospholipids has been observed in the prefrontal cortex in other AD studies^30^, possibly due to increased oxidative stress, which nonenzymatically degrades PUFA species^34^, potentially contributing to the reduced levels of PUFA in SAD patients. However, the mechanism underlying lower PUFA levels in AD patients remains unclear and the contribution of dietary PUFA to brain PUFA levels needs further study.

LPE 22:6 regiospecificity (sn-1 vs sn-2) of the glycerol backbone has not been specified in most prior studies and the physiological function of LPE might be regiospecific if not subject to regioselective enzyme-mediated transformation. Specifically, our study demonstrated that brain LPE 20:4 [sn-1] and LPE 22:6 [sn-1] showed significantly lower abundance in SAD than the control group. LPE is low in abundance in the brain and little is known regarding its relative changes in AD. In animal studies, brain LPE 20:4 and LPE 22:6 levels trend lower during aging^36^ or with impaired spatial cognitive abilities or memory^37^. Specifically, LPE 22:6 (omega-3) levels recover during the recovery of trauma brain injury^38^. In contrast, hippocampus LPE 18:1 and LPE 22:6 tend to be increased in vascular dementia^39^ and AD treatments for the inhibition of either PLA_2_^40^ or BACE1^41^ reduce LPE accumulation in brain. Even though the pathological significance of LPE is inconclusive in the brain, a few studies suggest the function of exogenous LPE species could stimulate neurite outgrowth in cell culture^42–44^. Hisano and others have reported the beneficial effect of LPE 18:1 [sn-1] on primary cortical neuron growth against glutamate-induced excitotoxicity via the activation of the GCPR-PLC-PKC-MAPK pathway, and compared to other phospholipids, LPE exhibited the most significant impact on neurite morphology as observed by an increase of microtubule associated protein 2-positive dendrites^42,43^. LPE isolated from *Grifola frondosa* mainly consists of LPE 18:2 [sn-1] exhibiting anti-apoptotic activity and enhancing neuronal differentiation through MAPK activation in PC-12 cells^44^. Moreover, in a mouse model, hepatic levels of LPE 22:6 [sn-1] are negatively associated with non-alcoholic steatohepatitis compared to control mice, and interestingly, highly unsaturated acyl chain in LPE shows a preference for the LPE [sn-1] form^45^. Taken together, this supports that the PUFA of LPE at its sn-1 position is potentially favorable for neuron function and lipid metabolism; however, more studies are needed to clarify roles of these species in brain pathology.

Our multi-omics analyzes revealed that three lipid co-expression modules consisting of either LPE only or a combination of LPE and LPC correlate more strongly to AD clinical traits. Particularly, the M4 lipid module, mainly driven by LPE 22:6 [sn-1] and LPE 20:4, was associated with the AD protein network modules MAPK/metabolism (black), post-synaptic density (green), and Cell-ECM interaction (greenyellow); these were highly associated with AD neuropathology and cognitive function. Johnson *et al*. (2022)^8^ demonstrated that the MAPK/metabolism module enriched in proteins co-localized to Aβ plaques and Tau neurofibrillary tangles, was strongly associated with cognitive decline, and trended towards enrichment for AD genetic risk. The green post-synaptic density module was associated with cognitive preservation and enriched in proteins positively correlated to cognitive resilience, while negatively correlated to tau microtubule-binding domain (MTBR) peptide levels. The cell-ECM interaction module was positively associated with MTBR and correlated to cognition decline prior to the adjustment of neuropathology. Both post-synaptic density and Cell-ECM interaction modules correlating to MTBR tended to be altered in AD. Although the physiological significance of LPE in brain function is rarely studied, Lee and others have demonstrated that LPE increases intracellular calcium influx in PC-12 neuron cells and SH-SY5Y neuroblastoma cells^46,47^ while LPE induces Calcium flux independent of LPAR^48^. Age-related alterations in neuronal calcium (Ca^2+^) largely contribute to AD pathology and studies of brain tissues have shown significant changes in levels of proteins or genes directly involved in neuronal Ca^2+^ signaling^49^. Briefly, Ca^2+^ dysregulation is associated with familial AD mutations such as in presenilin-1 (PSEN-1) and APP, and the disruption of Ca^2+^ handling is linked to Tau or Aβ accumulation^50^. Aβ interacts with Ca^2+^-related receptors (NMDAR, AMPAR) and channels (VGCC), and further induces Ca^2+^ excess influx to cytoplasm. Other unbalanced Ca^2+^ conditions in AD also include Ca^2+^ from endoplasmic reticulum (ER) leakage (RyR, IP3R, and SERCA), mitochondrial Ca^2+^ overload, and dysfunction of Ca^2+^ buffering proteins^49–51^. Calcium signaling plays an important role in synaptic plasticity triggering several kinase cascades such as calcium/ calmodulin-regulated protein kinases (CaMK), the cAMP-dependent protein kinase A (PKA), PKC, and MAPK/ERKs^51^. Although in our proteomic dataset, most of these calcium signaling-related proteins were not significantly different among these three AD phenotypes, proteins related to activation of NMDAR and post synaptic signaling transmission were significantly up-regulated in controls vs SAD including glutamate ionotropic receptor AMPA type subunit 1–3, GRIA1–3 (AMPAR), voltage-dependent calcium channel gamma-2–4, CACNG2–4 (VGCC), PKC alpha binding protein (PICK1), and Calcium/calmodulin-dependent protein kinase type IV (CAMK4) (Fig. S12c). APP and PSEN-1 double knockin mouse models demonstrate that a decrease of AMPAR efficacy is relevant to synaptic downscaling, an early onset phenomenon in AD, and AMPAR is crucial for long-term potentiation (LTP), critical for memory encoding as well as memory flexibility^52^. Moreover, treatment using electromagnetic fields (EMF) has shown that human plasma levels of LPE 20:4 and LPE 22:6 are increased after the exposure to high-voltage electric potential and an increase of LPE is possibly due to elevated hydrolysis by phospholipase A2 (PLA_2_)^53,54^. Mostly, EMF therapy has been reported to be associated with VGCC stimulation in various cell types^55^. Based on our results and literature, LPE species exhibit potential as therapeutic targets for AD via calcium homeostasis in brain.

In our study, a decreased ratio of LPE to PE was observed in SAD compared to controls, indicating possibly inactive hydrolysis of PE to LPE in SAD. PLA_2_ is responsible for catalyzing the hydrolysis of phospholipids to lyso-phospholipids and free fatty acids, and the reduction of the hippocampus group IVA isoform of PLA_2_ (PLA2G4A) has been shown to ameliorate Aβ-dependent deficits in a hAPP mouse model^56^. However, this PLA_2_ isoform was not detected in the DLPFC proteomic dataset reported by Johnson *et al*^8^ possibly due to differential expression in different brain regions. A slightly higher expression of PLA2G15 was observed in SAD (P = 0.064), while the protein levels of other PLA_2_ isoforms tended to be similar in controls versus SAD, but did not reach statistical significance. PLA2G15 acts as a lysosomal phospholipase preferring PC as substrate. In a PLA2G15 knockout mouse model, a nearly 2-fold increase in both PC and LPC levels in alveolar lavage fluid is observed^57^. Unlike the strong effects observed in knock out studies, the subtle change in brain PLA2G15 abundance in our proteomic data cannot fully reflect LPC/PC or LPE/PE ratio changes in our lipidomic analysis; however, the mechanism by which a decreased level of total LPE or specifically, LPE 22:6 [sn-1] occurs in SAD brain needs more study. In contrast, myelin disruption is associated with accumulated amyloid plaques^58^. Myelin lipid disruption is specific to the edges of the white matter in the corpus callosum, while no disruption of the myelin sheath is observed in gray matter. Together with myelin loss in white matter, depletion of PE and increased LPE are observed in a 5xFAD mouse model^58^. In addition to LPE release from PE through PLA_2_ hydrolysis, the uptake of plasma LPE can be regulated by the Sodium-dependent LPC symporter (Mfsd2a) and transferred to the brain through the blood brain barrier^59^. However, this is di cult to validate because Mfsd2a protein was not detected and we lack information about circulating LPE. Despite the complexity introduced by regional differences, cell types shift, and neuropathological conditions, changes in LPE levels associated with AD brain alterations has been highlighted in several AD studies^37,41,58,60^, indicating the significance of LPE in AD. Moreover, LPE metabolism has been reported being altered by obesity^61^ and by brown fat activity^62^, and notably, mid-life obesity links to a higher risk of dementia^63^. However, direct evidence regarding the role of LPE, specifically LPE 22:6 [sn-1], on AD pathology and the mechanism underlying LPE loss in AD requires further investigation.

In network analysis, the M4 module is strongly associated with the M3 module. Among the hub lipids in the M3 module, LPC 22:6 shares greater similarity with LPE 22:6 in the M4 module, both in terms of pathway and higher abundance in controls than the SAD group. At the lipid class level, lower LPC concentrations have been reported in the prefrontal cortex^64^, and frontal cortex^65^ of patients with AD compared to controls, aligning with our findings in the DLPFC in this study. DHA (C22:6) constitutes nearly 50% of PUFA content in the brain^66^, and depending on the brain regions, a characteristic of AD is the presence of lower DHA levels in brain phospholipids, ranging from about 15–60%^67^. However, dietary DHA, in the form of free DHA, is either resynthesized into TG or bound to albumin in the blood. The former is delivered to peripheral tissues through lipoprotein rather than the brain^68,69^, and the latter is released from albumin and transported along the outer membrane in BBB via passive diffusion^70^. In contrast, dietary LPC 22:6 is the brain’s preferred source of DHA through Mfsd2a^59^, which improves cognitive function compared free DHA supplements in a mouse study^68,69^.

Utilizing a proteomic dataset from iPSC-derived neurons^71^ from matched ROSMAP individuals, we found that LPE 22:6 is highly associated with Echinoderm microtubule-associated protein-like 4 (EML4), which is essential for the stabilization of microtubules (Fig. S13a). In line with our results, the lipid M4 module containing LPE 22:6 and LPE 20:4 correlates to a post-synaptic density module that is negatively associated with MTBR levels. According to Lagomarsono’s finding^71^, the protein levels of PPP1CA, a core catalytic component of protein phosphatase 1 (PP1), are significantly lower in the AD brain while PPP1R1A, a negative regulator of PP1, shows higher steady state levels. Aβ42/37 treatment in neuronal cells reduces PPP1CA protein levels and subsequently, influences tau phosphorylation and aggregation, indicating the contribution of Aβ-reduced PP1 activity to disturbed tau proteostasis. Interestingly, our correlation analysis (Fig. S13b-c) showed that LPE 22:6 has a negative correlation to PPP1R1A (cor=-0.58, p = 0.0015) and a positive correlation to PPP1CA (cor = 0.28, P = 0.16), suggesting a possible role of LPE 22:6 in AD development.

Our integrative multi-omics results revealed that LPE, including LPE 22:6 [sn-1], is strongly related to AD disease-associated protein modules such as the modules representing MAPK/metabolism, post-synaptic density, and cell-ECM interaction, implicating that LPE species may serve as promising therapeutic targets and even possibly as dietary supplementation for treatment of AD. These primary results provide future directions for studying the underlying mechanisms of LPE in AD brain and underscore the utility of future studies in cellular systems to uncover LPE-relevant neuronal biology in processes of different cell types contributing to AD pathologies.

## Methods

### Source of brain tissue samples and case classification

Brian tissues were from the dorsolateral prefrontal cortex (DLPFC) from 316 subjects in the ROSMAP cohort studies of aging and dementia^23^. All participants enrolled without known dementia and agreed to annual clinical evaluation and brain donation. Both studies were approved by an Institutional Review Board (IRB) of Rush University Medical Center. Participants signed an Anatomic Gift Act and both repository and informed consents. All clinical data, including sex, age, education, postmortem interval (PMI; refers to the interval between death and tissue preservation in hours), race, GlobalCogFunc average of standardized z-scores of 19 cognitive tests), CERAD, Braak, Reagan, ApoE Risk, gpath, amyloid plaques, and neurofibrillary tangles, were summarized in Table 1. All methods have been previously reported^72–74^. GlobalCogFunc was assessed longitudinally by Z-score of global cognitive performance averaged across 19 tests spanning 5 cognitive domains, including orientation, attention, memory, language, and perception. AD case classification (Diagnosis) in ROSMAP was defined as follows: cases with CERAD 0–1, and Braak 0–3 without dementia at last evaluation (if Braak = 3, then CERAD must equal 0.) were categorized as controls; cases with CERAD 1–3 and Braak 3–6 without dementia at last evaluation were categorized as AAD; cases with CERAD 2–3 and Braak 3–6 with dementia at last evaluation were categorized as SAD. Dementia was defined as Mini-Mental State Examination (MMSE) score < 24, Cognitive Abilities Screening Instrument (CASI) score < 81 or Clinical Dementia Rating (CDR) ≥ 1, and all definition of dementia is described in Balsis *et al*., 2015^75^.

### Sample preparation

Approximately 50 mg of gray matter dissected from DLPFC were added to a 96-well plate with 250 μl PBS and 1.4 mm ceramic beads, and homogenized for 4.0 m/s for 20 sec at 4°C. After homogenization, the samples were centrifuged at 4000 rpm for 10 min to pellet cell debris and the supernatant was extracted using a combination of Methyl tert-butyl ether (MTBE) and methanol using an automated sample handling manifold, Biotage Extrahera (Biotage, Uppsala, Sweden). For this, the homogenate was added to preconditioned 96-well plates, each well containing 10 μl internal standard obtained from Splash Lipidomix (Avanti Polar, Birmingham, AL). To each well, 200 μl methanol containing 50 μg/ml butylhydroxytoluene (BHT) was added. The sample was mixed by 3 up and down passes of the automated sample handling pipette arm. The samples were then centrifuged at 4000 rpm for 10 minutes to pellet precipitated protein. The supernatant was then transferred to a separate deep well 96-well plate. To this, 250 μl MTBE:methanol (3:1 v/v) was added and mixed by 3 up and down passes. The sample plate was then centrifuged at 1000*g for 3 minutes and the supernatant filtered through a 0.25 μm polytetrafluoroethylene (PFTE) filter plate (Biotage, ISOLUTE^®^ FILTER+, Uppsala, Sweden). The extract was then dried under nitrogen for lipidomic analysis. Dried lipids were reconstituted in 200 μl 1:1 acetonitrile: isopropanol for LC-MS/MS analysis.

### Non-targeted lipidomics analysis

Ten microliters of lipid extract were deposited on a Thermo Accucore C18 column on a Thermo Vanquish Ultimate 3000 UPLC coupled to a Thermo Orbitrap ID-X Tribrid mass spectrometer (Thermo, Waltham, MA). Chromatography was operated at a 0.4 ml/min flow rate at 40°C over a 15-minute gradient. The mobile phase of UPLC grade solvents consisted of solvent A: 10 mM ammonium formate in 60% acetonitrile with 0.1% formic acid and solvent B: 10 mM ammonium formate in 10% acetonitrile and 90% isopropanol with 0.1% formic acid. All chromatography parameters are shown in Table S1. Data were acquired in both positive and negative mode. Data were acquired at full scan mode at a resolution of 120,000 for all samples. Iterative data dependent acquisition (DDA) was collected on pooled samples at a resolution of 15,000 using step-wise collision energy adjustment to obtain substructure information enabling lipid identification. A table of all instrumental parameters is listed in Table S2. Raw data were processed using LipidSearch ver 4.2 (Thermo Fisher, San Jose, CA) and a total of 2200 lipids from both negative and positive modes were identified via full scan with data dependent MS^2^ (ddMS^2^)^20^. To focus our analysis and ensure data quality, we filtered the dataset to contain only lipids with a signal to noise ratio of greater than 3, a peak quality of greater than 0.6, and high MS/MS confidence identification (levels A, B and C in LipidSearch 4.2), resulting in 343 high confidence lipids (excluding internal standards) for further statistical analysis (Fig. S1).

### Lipid differential abundance analysis

Prior to differential abundance analysis, we normalized the lipidomics data to remove systematic variations based on quality control (QC) pool samples using the SERRF R package^76^. Log_2_ transformation was applied after normalization. The covariates age, gender, post-mortem interval (pmi), education, and race did not influence the lipidomic data assessed using the manova function in the dplyr R package (p > 0.05) so no covariance with these factors was removed by regression of the log_2_ post-normalized data. The final adjusted 343 lipids were used in downstream analysis. Differential lipid abundance was determined using one-way analysis of variance (ANOVA) followed by either Fisher’s least significant difference (LSD) test or Tukey post-hoc test across Control, AAD, and SAD cases. For two-group comparisons, Student’s t-test was used to determine if the means of two groups reached a significant difference (P < 0.05). The same comparison method was applied in proteomic datasets. Differential abundance is presented as violin plots using R package ggplot2^77^.

### Lipid set enrichment analysis (LSEA)

The Lipidr R package^78^ was used for visualizing log_2_ fold change of lipid chain length, unsaturation, and identifying significantly enriched lipid sets between two groups. Mirroring gene set enrichment analysis^79^, Lipidr grouped lipids to sets based on their lipid annotation, and then ranked individual lipids by their log_2_ fold changes, enrichment scores, and significance, which were calculated for each lipid set using an efficient permutation algorithm. To identify the lipid classes that were enriched within each WGCNA module, an over-representation analysis was performed using one-sided Fisher exact t-tests with all annotated lipids as the background. Hub lipids (module membership (MM) > 0.60) within each module were analyzed as the query. Raw p-values were adjusted within each module using the Benjamini Hochberg procedure and significantly enriched lipid classes were selected at a 5% false discovery rate (FDR).

### Weighted lipid co-expression network analysis

A weighted lipid co-expression network was built with R package WGCNA^80^. Briefly, one sample was excluded due to insufficient clinical information, and an outlier was removed based on its Euclidean distance in a sample clustering plot. Consequently, 314 cases were included for subsequent WGCNA analysis. A thresholding power of 8 was chosen (scale-free fit index, R^2^ = 0.958), and the signed network was generated by the component-wise minimum values for topologic overlap (TO). Lipid features were hierarchically clustered by distance measured by dissTom (1-TO) and initial lipid modules were assigned using the dynamic tree-cutting algorithm (Arguments: method =”tree”, cutHeight = 0.97, deepSplit = 3, and minModulesize = 2). After network analysis, 17 modules were found and the independence of each module was confirmed by T-distributed Stochastic Neighbor Embedding (t-SNE) analysis using Rtsne R package. The module eigenlipid values (MEs, the first principal component of a given module) available for each sample were correlated to case sample clinical and pathological traits, thereby calculating associations between modules and traits using the Pearson correlation method. Three modules significantly associated (P < 0.05) with all AD clinical traits (Diagnosis, GlobalCogFunc, Reagan, Braak, CERAD, global pathology, amyloid, and neurofibrillary tangle quantitation) were selected for further analysis.

### Integration of lipid and protein modules

The proteomic data of ROSMAP brain tissues were published in 2022^8^ and the module eigenprotein values calculated using WGCNA were obtained from our team. Sample-matched individuals (n = 266) with both proteomics and lipidomics were identified, and the input of clinical-pathological traits and ME summarization in both omics were further used to identify multi-omic biomarker panels that can discriminate the AD class using DIABLO (Data integration analysis for biomarker discovery on latent components) in the mixOmics R package^25^. The threshold criteria were set to correlation > 0.7 and the network was plotted using Cytoscape^81^.

### Omics-wide association analysis

To gain a holistic view of the relationship between selected lipids from lipid modules M3 and M4, and proteomics, we further conducted integrated network analysis using xMWAS^82^. Sample-matched individuals (n = 266) with both proteomics and lipidomics were identified, and the input of normalized values in both omics were used to calculate the pairwise associations between each protein and selected lipid species using partial least squares regression. The threshold criteria were set to correlation > 0.5 and P < 0.05 by Student T-test.

### GO enrichment (over representation) analysis

To characterize differentially expressed proteins based on gene ontology annotation, UniProt protein ID was converted to ENTREZ ID (Gene ID) using R packages, AnnotationDbi^83^ and org.Hs.eg.db^84^. To examine the cellular component, biological process, and molecular function of proteins associated with either selected lipid species or communities obtained from xMWAS, the gene products with absolute correlation coefficients over 0.5 were selected for GO enrichment analysis using clusterProfiler R package^85^.

### Other statistical analysis

The following plots and statistical analyses were performed using RStudio version 4.1.1^86^, unless otherwise specified. Supervised partial least-squares discriminant analysis (PLS-DA) to visualize the lipid difference between groups, the contributions of each variable from a group, the prediction background showing the prediction area by each group and area under the curve (AUC), and receiver operating characteristic curve (ROC) analysis for PLS-DA were calculated by mixOmics R package^26^. Heatmap showing the overall information for lipidomics and clinical traits; boxplots representing the median, maximum and minimum; volcano plot showing the log2 fold changes of lipid species and P value between groups; bubble heatmap representing correlations and P values; and chordDiagram plots representing the positive and negative correlations between selected variables were generated using the R packages pheatmap, RColorBrewer, ComplexHeatmap, ggplot2, ggrepel, stringi, ggcorrplot, reshape2, and circlize. The correlations were performed using biweight midcorrelation and Pearson correlation with Student asymptotic p values, and Kruskal-Wallis one-way ANOVA was used for analyzing the significant difference between groups; these are functions as implemented in WGCNA R package. The violin plots representing the median, the density of measured points by violin width, 25th /75th percentiles, lower/upper adjacent values, and outliers, the volcano plots showing the pairwise comparison of differential levels of lipid classes, and bar plots were generated by GraphPad prism 8.0.1.

## Figures and Tables

**Table 1:** Characteristics of overall brain samples by the three groups, Controls, AAD (Asymptomatic Alzheimer’s disease), and SAD (Symptomatic AD).

Clinico-pathologocal Factors	Controls	AAD	SAD
*Participants (n)*	92	77	147
*Age (years)*	86.44 ± 6.18^b^	89.92 ± 6.04^a^	91.17 ± 6.20^a^
*Gender*			
*Male*	35	15	39
*Female*	57	62	108
*Education (year)*	15.59 ± 3.58^a^	15.44 ± 3.53^a^	15.90 ± 3.41^a^
*Race*			
*White American*	89	75	144
*Black/African American*	2	2	2
*American Indian/Alaska Native*	0	0	1
*Unknown*	1	0	0
*PMI (hours)*	7.23 ± 3.75^a^	7.68 ± 5.17^a^	8.16 ± 4.98^a^
*GlobalCongFunc*	0.25 ± 0.46^a^	−0.09 ± 0.69^b^	−1.29 ± 1.08^c^
*Braak*	*2.48 ± 1.13* ^c^	*3.64 ± 0.74* ^b^	*4.24 ± 0.85* ^a^
*CEARD*	*3.61 ± 0.57* ^a^	*1.58 ± 0.50* ^b^	*1.48 ± 0.50* ^b^
*Reagan*	*3.03 ± 0.18* ^a^	*1.94 ± 0.25* ^b^	*1.71 ± 0.45* ^c^
*ApoE Risk*			
*2*	3	0	1
*1*	20	11	13
*0*	63	50	97
*1*	6	16	36
*Global AD pathology*	0.18 ± 0.24^c^	0.80 ± 0.38^b^	1.03 ± 0.55^a^
*B-Amyloid*	1.11 ± 1.98^b^	6.75 ± 4.33^a^	7.55 ± 4.52^a^
*Tangles*	2.14 ± 2.02^c^	3.94 ± 3.42^b^	7.99 ± 6.04^a^

Data present mean ± SD. Different superscript letter within the row indicates significant differences based on one-way ANOVA and LSD test at p < 0.05. Education level refers to the years of education which is based on the number of years of regular school reported at baseline cognitive testing. PMI, post-mortem interval refers to the interval between death and tissue autopsy in hours; other abbreviations or clinical traits were mentioned in the method section. Amyloid: Overall β-amyloid load from the average of 8 brain regions. Tangles: Tangle density from mean of 8 brain regions. GlobalCogFunc: Global cognitive function from average of 19 tests. gpath: Global burden of AD pathology based on 5 regions.

## Data Availability

ROSMAP resources can be requested at https://www.radc.rush.edu and www.synpase.org. Data available upon reasonable request.
